# Metabolic Alteration in Hepatocellular Carcinoma: Mechanism of Lipid Accumulation in Well-Differentiated Hepatocellular Carcinoma

**DOI:** 10.1155/2021/8813410

**Published:** 2021-02-18

**Authors:** Hideo Suzuki, Motoyuki Kohjima, Masatake Tanaka, Takeshi Goya, Shinji Itoh, Tomoharu Yoshizumi, Masaki Mori, Mariko Tsuda, Motoi Takahashi, Miho Kurokawa, Koji Imoto, Shigeki Tashiro, Akifumi Kuwano, Masaki Kato, Seiji Okada, Makoto Nakamuta, Yoshihiro Ogawa

**Affiliations:** ^1^Department of Medicine and Bioregulatory Science, Graduate School of Medical Sciences, Kyushu University, 3-1-1 Maidashi, Higashi-ku, Fukuoka 812-8582, Japan; ^2^Department of Gastroenterology, Kyushu Medical Center, National Hospital Organization, 1-8-1 Zigyohama, Chuo-ku, Fukuoka 810-8563, Japan; ^3^Department of Pathophysiology, Medical Institute of Bioregulation, Kyushu University, 3-1-1 Maidashi, Higashi-ku, Fukuoka 812-8582, Japan; ^4^Department of Surgery and Science, Graduate School of Medical Sciences, Kyushu University, 3-1-1 Maidashi, Higashi-ku, Fukuoka 812-8582, Japan; ^5^Graduate School of Nutritional Sciences, Nakamura Gakuen University, 5-7-1 Befu, Jounan-ku, Fukuoka 814-0198, Japan; ^6^CREST, Japan Agency for Medical Research and Development, 1-7-1 Otemachi, Chiyoda-ku, Tokyo 100-0004, Japan

## Abstract

**Objective:**

Metabolic alteration is widely considered as one of the hallmarks of cancer. Hepatocellular carcinoma (HCC) presents a unique pathological feature in which lipid accumulation is common in well-differentiated HCC and rare in poorly differentiated HCC; however, the underlying mechanism remains unclear.

**Methods:**

Tissue samples were obtained from 103 HCC patients who had undergone hepatic resection and 12 living donors of liver transplantation. We evaluated metabolic gene expressions in cancer tissues as well as background noncancer tissues and compared the expressions by the degree of cancer differentiation and by liver disease states. Besides, the metabolomics was evaluated and integrated to gene expressions in nonalcoholic steatohepatitis (NASH)-HCC model mice.

**Results:**

In cancer tissues, the expression levels of enzymes related to glycolysis, pentose phosphate pathway (PPP), and fatty acid (FA) synthesis were increased and that of tricarboxylic acid (TCA) cycle and *β*-oxidation were suppressed. Same metabolic alterations were observed in noncancer tissue as the liver disease progresses from healthy liver to chronic hepatitis, cirrhosis, and HCC. Similar alterations of metabolic genes were detected in NASH-HCC mice, which were consistent with the results of metabolomics. As the degree of cancer differentiation decreased, glycolysis and PPP were accelerated; however, FA synthesis and uptake were diminished.

**Conclusions:**

The metabolic alterations including glycolysis, PPP, TCA cycle, and *β*-oxidation became more prominent as liver disease progresses from normal, chronic hepatitis, cirrhosis, well-, moderately, and poorly differentiated HCC. FA synthesis and uptake were highest in well-differentiated HCC, which could explain the lipid accumulation.

## 1. Introduction

Hepatocellular carcinoma (HCC) is the sixth most common cancer [1] and the fourth most common cause of cancer-related deaths in the world [2]. HCC mostly develops in patients with chronic liver diseases related to viral (chronic hepatitis B and C), toxic (alcohol and aflatoxin), metabolic (diabetes, hemochromatosis, and nonalcoholic fatty liver disease), and immune (autoimmune hepatitis and primary biliary) factors [2]. Recent studies have shown that metabolic alterations are involved in the progression of HCC [3–5].

Metabolic alteration is widely considered as one of the hallmarks of cancer [6]. Due to uncontrollable growth, cancer cells need to modify their metabolism to facilitate the uptake and incorporation of nutrients into the biomass necessary to produce a new cell: amino acids for protein synthesis, nucleic acids for DNA duplication, and lipids for cell biomembrane synthesis. The well known metabolic change is the Warburg effect, in which cancer cells use aerobic glycolysis instead of mitochondrial oxidative phosphorylation for energy production, leading to increased lactate accumulation [7]. This ensures that glycolytic intermediates are not channeled directly into the tricarboxylic acid (TCA) cycle, but are instead diverted into anabolic pathways to provide macromolecular precursors such as pentose sugars, nucleotides, amino acids, and lipids. However, there have been few studies showing a comprehensive picture of glucose metabolism in HCC. Recent studies have also revealed the importance of lipid metabolic alteration in carcinogenesis [8]. Fatty acid (FA) functions as signaling molecules, storage compounds, and energy sources, as well as structural components of the cell membrane, all of which are essential for cancer cell proliferation. However, normal cells preferentially use exogenous circulating lipids, cancer cells, including HCC cells show a high rate of de novo FA synthesis [9]. Additionally, cellular uptake of FA is also increased in several types of cancers [10, 11].

It is known that hepatitis C virus (HCV) infection is closely associated with hepatocytic lipid metabolism. A recent study showed that lipid metabolism in HCV-infected liver was dysregulated; cholesterol and FA synthesis continued to increase without negative feedback [12]. As for nonalcoholic steatohepatitis (NASH), dysregulation of glucose and lipid metabolism occurs as early as in NASH, and continues throughout the entire oncogenic processes [13]. Moreover, a recent study reported that gene expression of glycolytic enzymes is upregulated in precancerous cirrhotic livers and is significantly associated with an elevated risk for developing HCC [14].

Besides, HCC is known to change the pathological features depending on the degree of differentiation. Among them, well-differentiated HCC is known to be a fat-containing tumor, and the accumulated fat disappears as the degree of differentiation becomes moderately or poorly differentiated. It is presumed that this fatty change of early-stage HCC tissue is caused by decreased portal vein flow and insufficient arterial development [15]; however, we sometimes encounter cases of hypervascular HCCs containing intracellular fat in the clinical practice. At this time, the metabolic profile of well-differentiated HCC and metabolic alteration, as the degree of differentiation becomes worse, are not fully clarified yet.

Here, we examined the gene expression levels of metabolic enzymes in cancer and noncancer tissues from human HCC samples. We also analyzed the expression in the progression of liver disease from normal liver, chronic hepatitis, cirrhosis, to HCC. In addition, we performed metabolomic analysis using NASH-HCC mouse model to validate that the gene expression results are actually linked to metabolomics. Furthermore, we compared the metabolic gene expressions by the degrees of HCC differentiation to investigate the mechanism of lipid droplet accumulation in well-differentiated HCC.

## 2. Methods

### 2.1. Patients and Samples

Tissue samples including cancer and noncancer parts were obtained from 103 patients with HCC who had received hepatic resection at Kyushu University Hospital. The pathologic differentiation grade of each HCC was assigned by two expert liver pathologists. As controls, normal liver tissues were obtained from 12 living donors of liver transplantation with normal liver function and histological findings. This study was approved by Kyushu University Hospital Ethics Committee (nos. 29–403 and 30–35), and written consent was obtained. The background characteristics of the HCC patients are shown in Table 1.

### 2.2. Animals

Male C57BL/6J wild-type (WT) mice were purchased from Japan SLC (Shizuoka, Japan). The melanocortin-4 receptor-deficient (MC4R–KO) mice on the C57BL/6J background were a generous gift from Dr. Joel K. Elmquist (University of Texas Southwestern Medical Center). All animals were acclimated to the environment in a temperature-, humidity-, and light-controlled room (12 h light and 12 h dark cycle) and allowed free access to water and a standard diet (CE-2; 343.1 kcal/100 g, 12.6% energy as fat; CLEA Japan), unless otherwise noted. Eight-week-old male MC4R–KO mice were fed a western diet (D12079 B; 468 kcal/100 g, 41% energy as fat, 34.0% sucrose, 0.21% cholesterol; Research Diets, New Brunswick, NJ) for 60 weeks, and male WT mice were fed the standard diet as control. At the end of the experiment, all animals were euthanized by isoflurane and the livers were harvested and immediately frozen in liquid nitrogen for mRNA extraction and metabolomic analysis. All studies were performed in accordance with the Guide for the Care and Use of Laboratory Animals (National Institutes of Health) and approved by the Animal Care Committee of Kyushu University.

### 2.3. Quantitative Reverse Transcription Polymerase Chain Reaction

Total RNA from liver tissue was extracted with TRIzol reagent (Invitrogen, Carlsbad, CA), and cDNA was synthesized with GeneAmp^TM^ RNA PCR (Applied Biosystems, Hammonton, NJ). Real-time PCR was performed using LightCycler FastStart DNA Master SYBR Green I (Roche, Basal, Switzerland). We measured mRNA expression levels of various metabolic genes: glucokinase (GK), glucose-6-phosphate dehydrogenase (G6PD), and pyruvate kinase (PK) as glycolysis-related genes, phosphoenolpyruvate carboxykinase (PEPCK) as gluconeogenesis, pyruvate dehydrogenase (PDH) *α*1, pyruvate dehydrogenase kinase (PDK) 1–4, aconitase (ACO) and isocitrate dehydrogenase (IDH) A1 as TCA cycle, acetyl-coenzyme A carboxylase (ACC) 1, fatty acid synthase (FAS), and sterol regulatory element-binding protein (SREBP) 1c as FA synthesis, diacylglycerol acyltransferase (DGAT) and peroxisome proliferator-activated receptor (PPAR) *γ* as triglyceride (TG) synthesis, hormone-sensitive lipase (HSL) as TG hydrolysis, microsomal triglyceride transfer protein (MTP) as TG secretion, and carnitine palmitoyltransferase (CPT) 1a, long chain acyl-coenzyme A dehydrogenase (LCAD), hydroxyacyl-coenzyme A dehydrogenase (HADH) *α*, and PPAR *α* as *β*-oxidation. To control variations in reactions, all PCR data were normalized against gene expression of ribosomal protein L32 (RPL32), TATA box binding protein (TBP), hydroxymethylbilane synthase (HMBS), and glyceraldehyde 3-phosphate dehydrogenase (GAPDH) as described previously [16]. The primer sequences used in this study are listed in Table S1 (see Supplementary Materials).

### 2.4. Metabolomic Analysis

Metabolomic analysis was performed by LSI Medience Corporation (Tokyo, Japan). In brief, the liver samples were homogenized using beads and suspended into 700 *μ*L distilled water and were mixed with methanol (2 mL) and chloroform (2 mL) for 10 min at room temperature. After centrifugation at 1000× *g* for 15 min, the supernatant was evaporated using nitrogen gas and dissolved with 10% acetonitrile aqueous solution (200 *μ*L). After adding internal standards, the samples were subjected to both liquid chromatography-mass spectrometry and capillary electrophoresis-mass spectrometry. A data file of mass spectrometry was converted to csv format with Agilent csv convertor. All peak positions (retention time and *m*/*z*) and areas were calculated using Markeranalysis (LSI Medience, Tokyo, Japan). All peak areas were aligned into one datasheet, and the errors of peak intensities were corrected using internal standards. Noise peaks were deleted after comparison with the peaks detected in blank samples. The metabolites were identified by comparing the retention times and *m*/*z* values with a standard dataset provided by LSI Medience Corporation.

### 2.5. The Statistical Analysis

Data were analyzed using JMP Pro Version 14 statistical software (SAS Institute Inc., Cary, NC, USA). The results were expressed as the means and standard deviation (SD) or standard error of the means (SE). The difference between means was analyzed by using Student's *t*-test. The value of *p* < 0.05 was considered statistically significant.

## 3. Results

### 3.1. The Metabolic Gene Expression Profiles in Cancer Tissues Relative to Background Noncancer Tissues

To investigate metabolic features in HCC, we isolated RNA from both cancer tissues and background noncancer tissues from HCC patients and compared the gene expression levels of metabolic genes.

**Figure 1:**
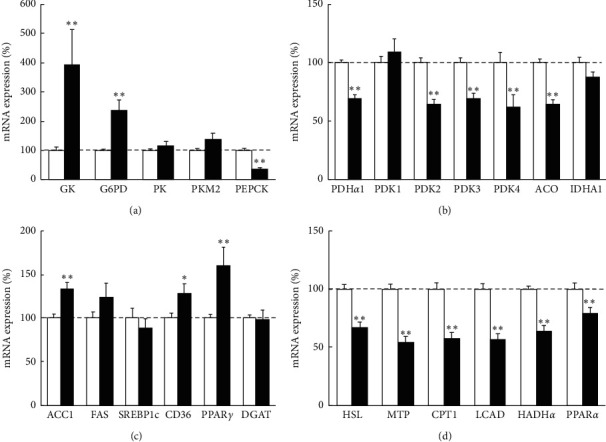
Metabolic gene expression in cancer tissues relative to noncancer tissues in human HCC samples. Quantitative RT-PCR analysis of metabolic genes related to (a) glucose metabolism, (b) pyruvate metabolism, tricarboxylic acid (TCA) cycle, (c) fatty acid (FA) synthesis and uptake, (d) triglyceride (TG) hydrolysis and secretion, and *β*-oxidation. The gene expression levels of cancer tissues were normalized to those of noncancer tissues and were presented as mean ± SE. ^*∗*^*p* < 0.05 and ^*∗∗*^*p* < 0.01 vs. noncancer tissue. GK, glucokinase; G6PD, glucose-6-phosphate dehydrogenase; PK, pyruvate kinase; PEPCK, phosphoenolpyruvate carboxykinase; PDH, pyruvate dehydrogenase; PDK, pyruvate dehydrogenase kinase; ACO, aconitase; IDH, isocitrate dehydrogenase; ACC, acetyl-coenzyme A carboxylase; FAS, fatty acid synthase; SREBP, sterol regulatory element-binding protein; PPAR, peroxisome proliferator-activated receptor; DGAT, diacylglycerol acyltransferase; HSL, hormone-sensitive lipase; MTP, microsomal triglyceride transfer protein; CPT, carnitine palmitoyltransferase; LCAD, long chain acyl-coenzyme A dehydrogenase; HADH, hydroxyacyl-coenzyme A dehydrogenase.

Initially, we examined the expression of genes involved in glucose metabolism (Figure 1(a)). mRNA expression of GK (a rate-limiting enzyme of glycolysis) and G6PD (a rate-limiting enzyme of the pentose phosphate pathway (PPP)) was significantly increased in the cancer tissue compared to noncancer tissue. The expression of PK and PKM2 (enzymes of glycolysis) was also modestly high in cancer tissue with no statistical significance. In contrast, the expression of PEPCK (a rate-limiting enzyme of gluconeogenesis) was significantly downregulated.

In the next, we analyzed the expression of enzymes related to the TCA cycle (Figure 1(b)). mRNA expression of PDH*α*1 (enzymes that convert pyruvate into acetyl-CoA), PDK2/3/4 (inhibitors of PDH), and aconitase (an enzyme of the TCA cycle) was significantly downregulated in the cancer tissue. Moreover, the expression of IDH1 (a rate-limiting enzyme of the TCA cycle) was also modestly decreased. Regarding the expression of genes involved in lipid metabolism, the transcription levels of ACC1 (a rate-limiting enzyme of lipogenesis), CD36 (a membrane protein that facilitates FA uptake), and PPAR*γ* (a regulator of hepatic lipogenesis) expression were significantly increased, and FAS expression was also modestly increased (Figure 1(c)). We did not detect significant alterations in the expression of SREBP1c (a master regulator of FA synthesis) and DGAT (a rate-limiting enzyme of triglyceride synthesis). Additionally, the expression levels of HSL (lipolytic enzyme), MTP (a rate-limiting enzyme in VLDL secretion), CPT1 (a rate-limiting enzyme involved in the transport of long-chain FA into mitochondrial matrix for *β*-oxidation), LCAD, HADH*α* (enzymes of *β*-oxidation), and PPAR*α* (a critical regulator of *β*-oxidation) were significantly lower in cancer tissue than noncancer tissue (Figure 1(d)).

### 3.2. The Metabolic Alteration as Liver Disease Progression

To investigate the metabolic alteration as liver diseases progress, we next analyzed mRNA expression of liver tissue of each disease state (57 chronic hepatitis (CH), 46 liver cirrhosis (LC), and 103 HCC), compared to 12 normal control (NC).

**Figure 2:**
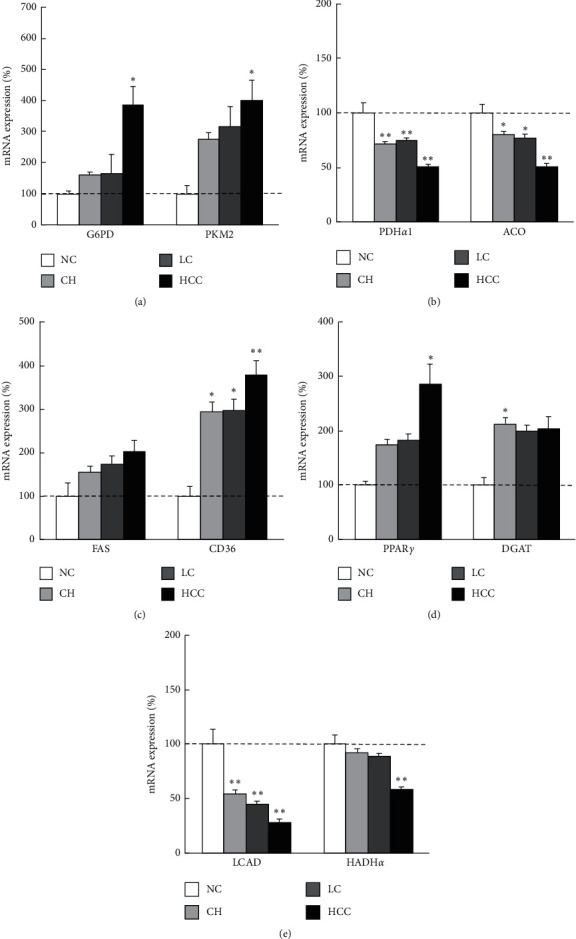
Metabolic gene expressions in each liver disease sate in human liver samples. Quantitative RT-PCR analysis of metabolic genes related to (a) glycolysis, (b) TCA cycle, (c) FA synthesis and uptake, (d) TG synthesis, and (e) *β*-oxidation, by liver disease states. The gene expression levels normalized to those of normal control tissues were presented as mean ± SE. ^*∗*^*p* < 0.05 and ^*∗∗*^*p* < 0.01 vs. normal control tissue. NC, normal control; CH, chronic hepatitis; LC, liver cirrhosis; HCC, hepatocellular carcinoma; G6PD, glucose-6-phosphate dehydrogenase; PK, pyruvate kinase; PDH, pyruvate dehydrogenase; ACO, aconitase; FAS, fatty acid synthase; PPAR, peroxisome proliferator-activated receptor; DGAT, diacylglycerol acyltransferase; LCAD, long chain acyl-coenzyme A dehydrogenase; HADH, hydroxyacyl-coenzyme A dehydrogenase.

Among the enzymes related to glucose metabolism, mRNA expression of G6PD and PKM2 was drastically increased in the order of NC, CH, LC, and HCC (Figure 2(a)). As for enzymes related to the TCA cycle, the expression level of PDH*α*1 and aconitase in the CH, LC, and HCC tissue was lower than that of NC, and that of HCC was the most significantly downregulated in all groups (Figure 2(b)). Regarding lipid metabolism, CD36, and PPAR*γ* expression were increased in the order of NC, CH, LC, and HCC (Figures 2(c) and 2(d)) and FAS expression was modestly increased in the same order. The expression levels of DGAT1 in the CH, LC, and HCC were higher than that of NC; there was no apparent difference among these three states (Figure 2(d)). About *β*-oxidation, the expression of LCAD and HADH*α* were decreased in the order of NC, CH, LC, and HCC (Figure 2(e)). The other genes involved in metabolic pathways also showed almost similar expression patterns.

### 3.3. Metabolomic Analysis and Its Association with Metabolic Gene Expression in NASH Mouse Model

To integrate metabolic gene expression results with metabolomics, we utilized the MC4R–KO mice that were mice models of human NASH [17, 18]. The mice fed high-fat diet showed a marked increase in body weight and liver weight after 60-week WD feeding. Their livers showed severe steatosis, ballooning degeneration, massive infiltration of inflammatory cells, and marked pericellular fibrosis and exhibited histological features of NASH (Figure S1B in Supplementary Materials). Moreover, multiple liver tumors were observed in all of the MC4R–KO mice examined (Figure S1C in Supplementary Materials), while the livers from WT mice fed the standard diet presented mild steatosis with no tumor (Figure S1A in Supplementary Materials).

**Figure 3:**
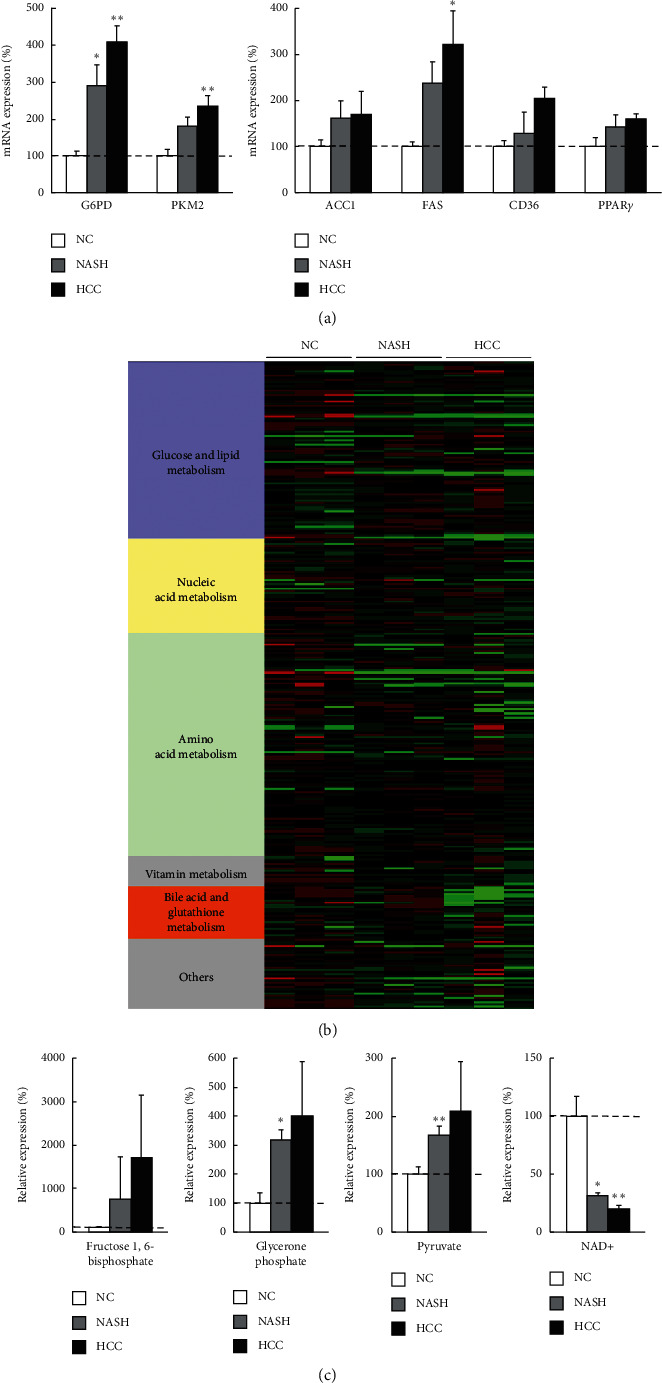
Gene expression analysis and metabolomics of liver samples from mouse NASH-HCC model. (a) Quantitative RT-PCR analysis of metabolic genes related to glycolysis, FA synthesis and uptake, and TG synthesis in cancer tissues (HCC) and noncancer tissues (NASH) from melanocortin-4 receptor-deficient (MC4R–KO) mice fed a western diet for 60 weeks. The gene expression levels were normalized to those of wild-type (WT) mice as normal control (NC) and were presented as mean ± SE. ^*∗*^*p* < 0.05 and ^*∗∗*^*p* < 0.01 vs. NC. (b) Heat map analysis of metabolomics in NC, NASH, and HCC. It was generated by coloring the values of all data across their value ranges. The color red demonstrated that the relative content of metabolites is high and green demonstrates that they are low. The brightness of each color corresponded to the magnitude of the difference when compared with the average value. (c) The amounts of glycolysis-related metabolites and NAD^+^ in NASH and HCC normalized to those in NC were presented as mean ± SD. ^*∗*^*p* < 0.05, ^*∗∗*^*p* < 0.01 vs. NC. NASH, nonalcoholic steatohepatitis; HCC, hepatocellular carcinoma; G6PD, glucose-6-phosphate dehydrogenase; PK, pyruvate kinase; ACC, acetyl-coenzyme A carboxylase; FAS, fatty acid synthase; SREBP, sterol regulatory element-binding protein; PPAR, peroxisome proliferator-activated receptor; NAD, nicotinamide adenine dinucleotide.

We examined metabolic gene expression in cancer tissues (HCC), noncancer liver tissues (NASH) from MC4R–KO mice, and normal liver tissue from WT (NC). Concerning glycolytic enzymes, mRNA expression levels of G6PD and PKM2 were upregulated in the order of NC, NASH, and HCC. Regarding lipogenesis-related enzymes, FAS expression was increased significantly in the order of NC, NASH, and HCC, and the expression of ACC1, CD36, and PPAR*γ* was also increased modestly in the same order (Figure 3(a)). These mRNA expression patterns were quite similar to that of the human samples. The other metabolic genes in the liver of MC4R–KO mice did not present definitive change.

We next analyzed the levels of the metabolites extracted from each tissue. The heatmap of metabolomics is shown in Figure 3(b). The levels of glycolysis-related metabolites including glycerone phosphate and pyruvate were increased drastically in the order of NC, NASH, and HCC, and fructose-1,6-bisphosphate was also increased modestly in the same order (Figure 3(c)). On the other hand, the level of nicotinamide adenine dinucleotide (NAD)+ was decreased in the order of NC, NASH, and HCC (Figure 3(b)).

### 3.4. The Metabolic Alteration by the Degree of HCC Differentiation

We examined metabolic gene expression levels of HCC relative to noncancer tissue and compared by the degrees of differentiation (20 well-differentiated, 57 moderately differentiated, and 26 poorly differentiated HCC).

**Figure 4:**
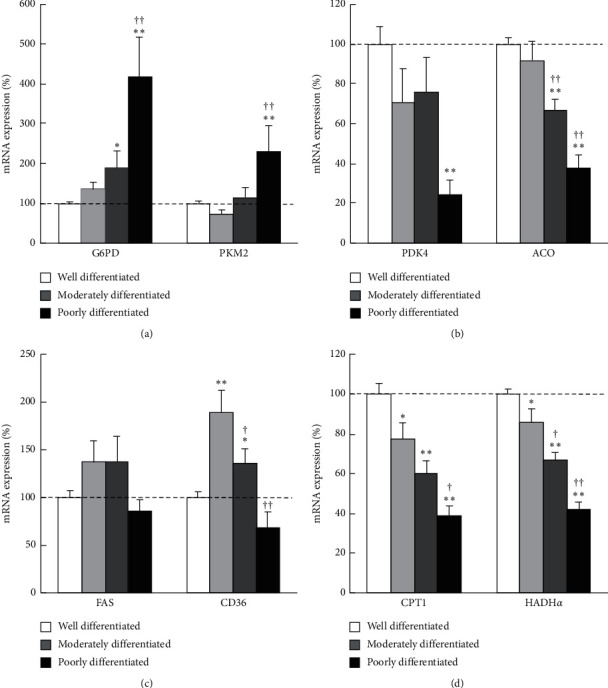
Metabolic gene expression levels in each degree of cancer differentiation in human HCC samples. Quantitative RT-PCR analysis of metabolic genes related to (a) glycolysis, (b) TCA cycle, (c) FA synthesis and uptake, and (d) *β*-oxidation. The gene expression levels normalized to those of noncancer tissues and were presented as mean ± SE. ^*∗*^*p* < 0.05 and ^*∗∗*^*p* < 0.01 vs. noncancer tissue. ^†^*p* < 0.05, ^††^*p* < 0.01 vs. well-differentiated HCC tissue. G6PD, glucose-6-phosphate dehydrogenase; PK, pyruvate kinase; PDK, pyruvate dehydrogenase kinase; ACO, aconitase; FAS, fatty acid synthase; CPT, carnitine palmitoyltransferase; HADH, hydroxyacyl-coenzyme A dehydrogenase.

Among the enzymes involved in glucose metabolism, mRNA expression of G6PD and PKM2 was significantly increased in the order of well-, moderately, and poorly differentiated HCC (Figure 4(a)). As for enzymes related to the TCA cycle, mRNA expression of PDK4 was significantly downregulated in poorly differentiated HCC tissue and that of aconitase was decreased in the order of well-, moderately, and poorly differentiated HCC (Figure 4(b)). Regarding the expression of genes involved in lipid metabolism, mRNA expression of FAS was modestly higher and that of CD36 was significantly higher in well-differentiated HCC tissue compared to that in noncancer tissue (Figure 4(c)). Interestingly, in poorly differentiated HCC, these expression levels were significantly suppressed and became lower than that of noncancer tissue. Concerning *β*-oxidation-related genes, the expression of CPT1 and HADH*α* was also decreased in the order of well-, moderately, and poorly differentiated HCC (Figure 4(d)). We checked the other metabolic genes and those also showed almost similar expression patterns.

## 4. Discussion

At first, we analyzed the metabolic status of HCC in comparison to background noncancer tissue and found upregulation of glycolysis, PPP, and FA synthesis and downregulation of the TCA cycle and *β*-oxidation in cancer tissue. As previously described, cancer cells rely on the aerobic glycolysis pathway instead of mitochondrial oxidative phosphorylation to achieve robust cellular proliferation [7]. In this study, the expression level of GK in the cancer tissue was upregulated (Figure 1(a)), which indicates an acceleration of glycolysis. Although a previous report showed that GK expression was downregulated in advanced HCC [19], this discrepancy could be due to the wide distribution of cancer differentiation including early-stage HCC in our study and the different background of enrolled patients. Another glycolytic enzyme, PKM2, is reported to be overexpressed in multiple cancer types [20–23] and that overexpression has been reported as a predictor of poor prognosis in diverse malignancies [24, 25] including HCC [26]. Here, the PKM2 expression level in noncancer tissue might have already increased with the liver disease progression, and the upregulation of that in cancer tissue compared to background noncancer tissue was limited. Compared to normal liver tissue, PKM2 expression of cancer tissue was significantly upregulated (Figure 2(b)). As opposed to upregulated glycolysis, TCA cycle-related enzyme was downregulated in HCC (Figure 1(a)). These findings suggested that Warburg effect, in which cancer cells use aerobic glycolysis instead of mitochondrial oxidative phosphorylation for energy production, was also identified in HCC tissue. The expression of G6PD, an enzyme involved in PPP, was also upregulated in the cancer tissue (Figure 1(a)). PPP acceleration in cancer tissue was also reported in previous reports [27, 28], and it is assumed that PPP supports cancer cell survival and growth by generating ribose for nucleic acid synthesis and nicotinamide-adenine dinucleotide phosphate (NADPH) necessary for FA synthesis and cell survival under stress conditions [29]. The expression of PDK4 was downregulated (Figure 1(b)), while FA synthesis was upregulated in HCC (Figure 1(c)). PDK4 predominantly inhibits pyruvate oxidation in the liver. Previous reports showed that PDK4 mediated the metabolic switch from glucose metabolism to fatty acid metabolism [30], and the lower expression of PDK4 increased lipogenesis in cancer tissue [31]. It is possible that acetyl-CoA converted from pyruvate via glycolysis upregulation and PDK4 downregulation was used for de novo FA synthesis instead of entering the TCA cycle in HCC cells. Additionally, FA uptake was increased, while *β*-oxidation and extracellular secretion of TG was suppressed in cancer tissue (Figures 1(c) and 1(d)). These mechanisms which increase FA in cancer cell could help the biosynthesis and modification of the lipid bilayer membrane in newly generated cancer cells [32].

**Figure 5:**
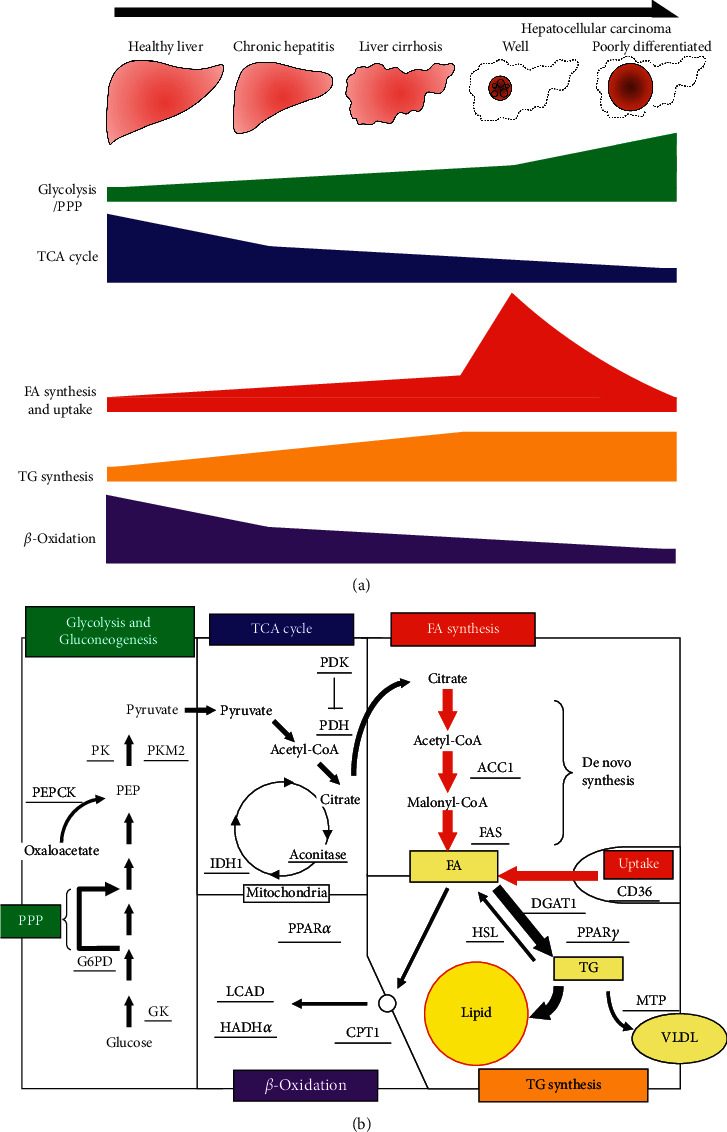
Schematic figures of metabolic alterations in HCC. (a) The schematic figure showing the metabolic alteration as liver disease progresses from normal liver, chronic hepatitis, cirrhosis, to well- and poorly differentiated HCC. Glycolysis and pentose phosphate pathway (PPP) were increased and the TCA cycle and *β*-oxidation were decreased throughout the disease progression course. FA synthesis and uptake were gradually increased until HCC development, while those of well-differentiated HCC were the highest and decreased as the degree of differentiation progressed. (b) Comprehensive schematic figure showing the metabolic features in the well-differentiated HCC. The thick arrows represent increased biochemical reactions indicated by gene expression analysis. GK, glucokinase; G6PD, glucose-6-phosphate dehydrogenase; PEP, phosphoenolpyruvate; PEPCK, phosphoenolpyruvate carboxykinase; PK, pyruvate kinase; PDH, pyruvate dehydrogenase; PDK, pyruvate dehydrogenase kinase; IDH, isocitrate dehydrogenase; ACC, acetyl-coenzyme A carboxylase; FAS, fatty acid synthase; PPAR, peroxisome proliferator-activated receptor; DGAT, diacylglycerol acyltransferase; HSL, hormone-sensitive lipase; MTP, microsomal triglyceride transfer protein; CPT, carnitine palmitoyltransferase; LCAD, long chain acyl-coenzyme A dehydrogenase; HADH, hydroxyacyl-coenzyme A dehydrogenase.

Next, we analyzed the metabolic alteration as liver disease progresses from normal liver, chronic hepatitis, cirrhosis, to HCC. We found that gene expression of enzymes in glycolysis, PPP and FA, and TG synthesis was increased (Figures 2(a), 2(c), and 2(d)) and that of the TCA cycle and *β*-oxidation was decreased with the progression of liver disease (Figures 2(b) and 2(e)). This series of metabolic alterations show similar patterns to the ones that have been confirmed in cancer cells compared to noncancer cells as mentioned above. These findings suggest that the metabolic alteration does not occur abruptly at the stage of carcinogenesis, but accumulates continuously as liver diseases progress (Figure 5(a)).

We could not analyze protein expression levels because of the limited amounts of human liver samples. Recently, *in vitro* human proteomic analysis showed that metabolic alteration including the Warburg effect was also seen in the cancer cells in the protein level as well as in mRNA level [33]. Alternatively, we performed metabolomic analysis and integrated it to mRNA expression analysis, using the animal model of NASH-HCC MC4R-KO mice. We found glycolysis-related gene expression and intermediate metabolites were also increased in HCC (Figures 3(a) and 3(b)). Besides, FA synthesis-related gene expression was also upregulated (Figure 3(a)), but unfortunately, metabolites involved in the TCA cycle and FA metabolism were too few to be detected by metabolomics. These data of the mice model supported the findings of metabolic alteration seen in human HCC.

Finally, we evaluated the metabolic alteration of HCC by the degree of differentiation and found that upregulation of glycolysis and PPP and downregulation of the TCA cycle and *β*-oxidation became more pronounced as the degree of differentiation progresses to moderately and poorly differentiated (Figures 4(a), 4(b), and 4(d)). On the other hand, it was quite surprising that FA synthesis and uptake were increased in well-differentiated HCC compared to noncancer tissue and decreased as the degree of differentiation progressed (Figure 4(c)). These novel findings could explain the reason why lipid droplets which are frequently observed in well-differentiated HCC are relatively rare in moderately and poorly differentiated HCC (Figures 5(a) and 5(b)). Although a previous study suggested that lipid droplets in well-differentiated HCC are caused by hypoxia due to insufficient blood flow [12], it might be due to a series of the metabolic alterations as liver disease progression.

Here, we analyzed the gene expression levels of the wide variety of etiology for HCC patients. In liver disease states associated with HCV or NASH, a similar metabolic alteration has been reported previously [12–14]. Because HCV and NASH-related samples represented a majority in this study, the findings could be strongly affected by the results from the tissue of HCV or NASH patients. However, HBV-related samples also showed similar gene expression patterns with HCV- and NASH-HCC (data not shown), which indicates the metabolic alterations might not be subjected to the cause of liver disease. Although we could not examine the protein expressions, the metabolic alternations in HCC were confirmed by metabolomic analysis using a mice model of NASH-HCC.

In summary, our gene expression analyses of human liver samples revealed that metabolic alterations which include upregulation of glycolysis, PPP, FA synthesis and uptake and downregulation of the TCA cycle and *β*-oxidation became more prominent as liver disease progresses from normal liver, chronic hepatitis, cirrhosis, to HCC. The result of metabolomic analysis using a mouse model of NASH-HCC was consistent with these findings. Same metabolic alterations except for FA synthesis and uptake became more pronounced as the degree of differentiation progresses to moderately and poorly differentiated HCC. Surprisingly, FA synthesis and uptake were highest in well-differentiated HCC and decreased as the degree of differentiation progressed, which could explain the unique histological feature, i.e., lipid droplet accumulation, of well-differentiated HCC. Although the defined mechanism of the alteration is still unclear, several compounds including statins, which were known to affect the metabolic pathway, suppressed the onset and growth of HCC [34, 35]. Moreover, recent studies showed that some specific inhibitors of metabolic enzymes, such as PKM2, ACC1, or SREBP1c, inhibited HCC progression [36–39]. The metabolic pathway could be a new therapeutic target for chemoprevention of HCC, although further studies should be investigated.

## Figures and Tables

**Table 1:** Clinical characteristics of the patients with HCC.

Number	103
Age (years)	68.1 ± 7.8
Gender (M/F)	79/24

*Etiology*	
(HBV/HCV/HBV + HCV/NBNC)	**15/65/2/21**

*Liver disease states*	
(Chronic hepatitis/liver cirrhosis)	**57/46**

*Cancer cell differentiation*	
(Well-/moderately/poorly differentiated)	**20/57/26**

*Paraclinical status*	
Alb (g/dl)	**3.95** **±** **0.44**
T-Bil (mg/dl)	**0.91** **±** **0.58**
AST (IU/l)	**51.4** **±** **41.8**
ALT (IU/l)	**44.9** **±** **31.5**
GGT (IU/l)	**84.8** **±** **96.9**
ALP (IU/l)	**317.3** **±** **130.3**
LDH (IU/l)	**211.2** **±** **71.4**
TC (mg/dl)	**168.9** **±** **43.3**
TG (mg/dl)	**109.0** **±** **56.6**
FBS (mg/dl)	**107.8** **±** **42.9**

Counts are presented as N; continuous data are presented as mean ± SD. Alb, albumin; T-Bil, total bilirubin; AST, aspartate aminotransferase; ALT, alanine aminotransferase; GGT, gamma-glutamyl transpeptidase; ALP, alkaline phosphatase; LDH, lactate dehydrogenase; TC, total cholesterol; TG, triglyceride; FBS, fasting blood sugar; HBV, hepatitis B virus; HCV, hepatitis C virus; NBNC, non-HBV and non-HCV.

## Data Availability

The clinical data used to support the findings of this study are included within the article.

## Supplementary Materials

Supplementary Materials The list of primers used in the present study is shown in Table S1. Microscopic images of the liver from WT and MC4R–KO mice are shown in Figure S1. Table S1: primers used in the present study.
