# Supplementary material for: Metabolic Alteration in Hepatocellular Carcinoma: Mechanism of Lipid Accumulation in Well-Differentiated Hepatocellular Carcinoma
Source: Can J Gastroenterol Hepatol. 2021 Feb 18;2021:8813410. doi: 10.1155/2021/8813410 (PMC7910064; doi:10.1155/2021/8813410)
Supplement: Supplementary Materials — The list of primers used in the present study is shown in Table S1. Microscopic images of the liver from WT and MC4R–KO mice are shown in Figure S1. Table S1: primers used in the present study. [file 8813410.f1.docx]

Table S1: Primers used in the present study

| **Genes** | **Forward (5'-3')** | **Reverse (5'-3')** |
| --- | --- | --- |
| **Human primers** | | |
| TBP | TGCTGCGGTAATCATGAGGATA | TGAAGTCCAAGAACTTAGCTGGAA |
| HMBS | AACAGCCCAAAGATGAGAGTGATTC | AATGTTGCCACCACACTGTCC |
| RPL32 | GATGCCCAACATTGGTTATGGA | CACGATGGCTTTGCGGTTC |
| GK | GAGTATGACCGCCTGGTGGA | CATGTACTTGCCACCTATGAGCTTC |
| G6PD | GGAGGGCGACGACGACGAAG | TCGGGCAGAAGGCCATCCCG |
| PK | CGTGAACCTCCAGAAGCCATC | TGTCACCACAATCACCAGGTCTC |
| PKM2 | AAGGCATCTGATGTCCATGAAGTTA | TCTCAATGCCTAGATCACCACGA |
| PEPCK | GGCATTATCTTTGGAGGCCGTAG | GCCAGGTATTTGCCGAAGTTGTAG |
| PDHα1 | GCTGGGATTGCTCTAGCCTGTAAG | TCCACAAAGCTGCCATGTTGTAA |
| PDK1 | CATGTCACGCTGGGTAATGAGG | GAGGTCTCAACACGAGGTCTTGG |
| PDK2 | GAAGGCTTTGGGACCGATG | CGCCAGGCTGACTTGTTGTAG |
| PDK3 | CCTTCAGTGGGATTGGTTCAGAG | GCCATTGTAGGAACCACATCATTG |
| PDK4 | CCACATTGGAAGCATTGATCCTAAC | TGATCACAGAGCATCCTTGAACAC |
| ACO | GAAACAGTCCTGCTGCTCGCTAC | GAGCCATAGGAGTTGAATTCTCGTG |
| IDH1 | AATCAGTGGCGGTTCTGTGGTAG | TGCAGCATCCTTGGTGACTTG |
| ACC1 | GAGGGCTAGGTCTTTCTGGAAG | CCACAGTGAAA TCTCGTTGAGA |
| FAS | AGCTGCCAGAGTCGGAGAAC | TGTAGCCCACGAGTGTCTCG |
| SREBP1c | GCGGAGCCATGGATTGCAC | CTCTTCCTTGATACCAGGCCC |
| CD36 | TCAATAGCGTCTGCAAATGGA | TTCAGAGATAACCATTGCGTGATAG |
| DGAT1 | AGCAACTACCGTGGCATCCTG | AATAACCGGGCATTGCTCAAGA |
| PPARγ | TGGAATTAGATGACAGCGACTTGG | CTGGAGCAGCTTGGCAAACA |
| HSL | TACCGCAGCCTAGTGCACAC | AGATGGTCTGCAGGAATGGC |
| MTP | AGCACCTCAGGACTGCGAAGA | CAGAGGTGACAGCATCCACCA |
| CPT1 | TTCACTGAGCACGGCAAGATG | GCAGCGATGTCTGGAAGCTGTA |
| LCAD | GTGTAACCCGAGCATTTGTGGAC | GCAATTGGGTACTCCCACATGTATC |
| HADHα | AGATGCAGCTGCTGGAGATTATCA | GGCGCAAGACACCTGGTAGTATAGA |
| PPARα | TCGGCGAGGATAGTTCTGGAAG | ACCACAGGATAAGTCACCGAGGA |
| **Mouse primers** | | |
| GAPDH | TGTGTCCGTCGTGGATCTGA | TTGCTGTTGAAGTCGCAGGAG |
| G6PD | CCGGAAACTGGCTGTGCGCT | CCAGGTCACCCGATGCACCC |
| PKM2 | AGATCCGAACTGGGCTCATCA | TAGATCTTGCTGCCCACTTCCAC |
| ACC1 | TGAGATTGGCATGGTAGCCTG | C​​TCGGCCATCTGGATATTCAG |
| FAS | TCCTGGAACGAGAACACGATCT | GAGACGTGTCACTCCTGGACTTG |
| CD36 | GATGACGTGGCAAAGAACAG | TCCTCGGGGTCCTGAGTTAT |
| PPARγ | AAGAGCTGACCCAATGGTTG | ACCCTTGCATCCTTCACAAG |

TBP, TATA box binding protein; HMBS, hydroxymethylbilane synthase; RPL32, ribosomal protein L32; GK, glucokinase; G6PD, glucose-6-phosphate dehydrogenase; PK, pyruvate kinase; PEPCK, phosphoenolpyruvate carboxykinase; PDH, pyruvate dehydrogenase; PDK, pyruvate dehydrogenase kinase; ACO, aconitase; IDH, isocitrate dehydrogenase; ACC, acetyl-coenzyme A carboxylase; FAS, fatty acid synthase; SREBP, sterol regulatory element-binding protein; DGAT, diacylglycerol acyltransferase; PPAR, peroxisome proliferator-activated receptor; HSL, hormone-sensitive lipase; MTP, microsomal triglyceride transfer protein; CPT, carnitine palmitoyltransferase; LCAD, long chain acyl-coenzyme A dehydrogenase; HADH, hydroxyacyl-coenzyme A dehydrogenase; GAPDH, glyceraldehyde-3-phosphate dehydrogenase.

A

B C

Figure S1: Histology of the liver from WT mouse fed a standard diet and MC4R-KO mouse fed a western diet for 60 weeks. Microscopic pictures of hematoxylin and eosin staining of normal liver tissue in WT mouse (A), background non-cancer tissue (B) and cancer tissue (C) in MC4R-KO mouse. Same as human NASH, fat deposition, inflammatory cell infiltration, and ballooning hepatocytes were observed (B). WT, wild type; MC4R-KO, melanocortin-4 receptor-deficient; C, cancer; NC, non-cancer. Original magnification; x200. Scale bar; 50μm.
